# Bone apposition at the mandibular angles as a radiological sign of bruxism: a retrospective study

**DOI:** 10.1186/s12903-021-01804-9

**Published:** 2021-10-18

**Authors:** Jens Christoph Türp, Michelle Simonek, Dorothea Dagassan

**Affiliations:** 1 Division of Temporomandibular Disorders and Orofacial Pain, Department of Oral Health & Medicine, University Center for Dental Medicine Basel (UZB), University of Basel, Basel, Switzerland; 2 Department of Oral Surgery, University Center for Dental Medicine Basel, University of Basel, Basel, Switzerland; 3 Center for Dental Imaging, University Center for Dental Medicine Basel, University of Basel, Basel, Switzerland

**Keywords:** Bruxism, Biological adaptation, Bone remodeling, Mandible, Panoramic radiography

## Abstract

**Background:**

The main objective of this investigation was to determine on panoramic radiographs the prevalence of macroscopically visible alterations (bone apposition in combination with directional change) in the mandibular angle region in bruxism patients. Another aim was to describe and detect different morphological characteristics of the jaw angles.

**Methods:**

Two hundred panoramic radiographs were studied: 100 images of adults with clinically diagnosed bruxism (73 women, 27 men, age range 21–83 years), 100 images of a comparison group consisting of adolescents (66 girls, 34 boys, age range 12–18 years).

**Results:**

The morphological changes of the 400 jaw angles could be classified into four degrees. In the adult group, almost half of mandibular angles showed bone apposition. Conversely, the prevalence in the control group was zero. The localization of the appositions corresponds to the insertions of the masseter and medial pterygoid muscles at the mandibular angle.

**Conclusions:**

The bone apposition at the mandibular angles should be interpreted as a functional adaptation to the long-term increased loads that occur during the contraction of the jaw closing muscles due to bruxism. Hence, radiologically diagnosed bone apposition may serve as an indication or confirmation of bruxism.

## Background

With an estimated prevalence of approximately 30% and 15% for awake and sleep bruxism [1], respectively, these masticatory muscle activities are a clinically significant phenomenon in the adult population. Jaw closure, which is a prerequisite for the execution of jaw clenching and tooth grinding in the context of bruxism, is caused by three paired masticatory muscles: the temporal, the masseter, and the medial pterygoid muscle. The latter two, which attach their tendons to the tuberosities of the mandibular angle, contribute to about 65% of the intrinsic strength of the jaw-closing muscles [2].

The vertical masticatory forces exerted during the first mastication cycles and measured directly on individual teeth range from 20 to 150 N depending on the food texture, with maximum forces of up to 250 N measured in single cases (chewing gummy bears). In contrast, the maximum voluntary biting forces between antagonistic molars are usually between 500 and 700 N [3]. However, the force exerted during sleep bruxism can significantly exceed the amplitude of the maximum voluntary bite force during wakefulness [4].

Sustained bruxism may have clinical consequences by increasing the risk of developing clinical signs and symptoms, such as tooth wear [5], fatigue [6, 7], pain of the masticatory muscles [7, 8] or temporomandibular joints (TMJs) [9, 10], anterior disk displacement [8] and TMJ clicking [6, 11], or masseter hypertrophy [12]. Current strategies for assessing the presence of bruxism are based on (a) self-report by the individual, (b) clinical examination, and (c) instrumental approaches such as electromyographic recordings, including polysomnography and/or audio/video recordings [13]. Together, they form a grading system with increasing likelihood of a valid diagnosis of bruxism. In contrast to anamnestic information and data obtained from the clinical examination, however, instrumental diagnostic procedures have the disadvantage that they are less frequently available, their use is associated with cost and time, and not every patient gives consent for this type of assessment.

In this situation, a previously neglected additional diagnostic observation may come into play: The flattening of the formerly rounded surfaces of the mandibular condyle and the posterior slope of the articular eminence. While this radiological sign has traditionally been associated almost exclusively with osteoarthrosis or osteoarthritis, it may also be the biological result of adaptive remodeling due to repetitive mechanical loading from compressive forces (e.g., jaw clenching) and thus represent nothing more than bony adaptation [14], due to the intimate relationship between function and form in biologic systems [15, 16]. In fact, already in 1939 Molnár [17] stated that “[t]he masticatory system is certainly subjected to the greatest stress by the masticatory musculature […] The effects of this stress, which can be seen in a functional shaping according to the law of ‘functional adaptation,’ can be demonstrated particularly well in the lower jaw bone […]”.

On closer examination of panoramic radiographs, we noticed that an appreciable number of individuals diagnosed with bruxism had bony changes not only in the condylar area, but also in another part of the mandible, the mandibular angle. Here, an increase in bone apposition was often observed. Since, to our knowledge, no study has been conducted on that topic, we wanted to investigate whether the prevalence of these morphological alterations is higher among bruxers compared to a control group. Another aim was to classify the degree of these morphological changes.

## Methods

### Study material

A total of 200 existing panoramic radiographs from two defined study groups were examined: One hundred radiographs from a group of adults with a clinical diagnosis of bruxism (made by JCT) from the Department of Oral Health & Medicine (73 women, 27 men; median age: 47.7 years, range 21–83 years) and 100 radiographs from a comparison group consisting of adolescents with completed orthodontic therapy from the patient pool of the Department of Pediatric Oral Health and Orthodontics (66 girls, 34 boys; median age: 14.3 years, range 12–18 years). The comparison group was composed of adolescents to determine the standard variants of mandibular angle morphology as a basis for further classification. It was assumed that no bone apposition had yet occurred in this age group.

All radiographs had been taken between May 2010 and May 2017 at the Clinic for Oral Surgery, Oral Radiology and Oral Medicine, School of Dentistry (now: Center for Dental Imaging) using the direct digital panoramic and cephalometric system Cranex D (Soredex, Tuusala, Finland; magnification factor: 1:1.25). The images were visualized with viewing software without applying further filter functions (Digora Version 2.9, Soredex by Kavo Kerr Group).

### Exclusion criteria

Exclusion criteria were subjects with a history of known metabolic bone disorders or a malignant tumor, as well as images with motion artifacts and strong ventral or retroflexion [18]. (In some patients with multiple panoramic radiographs, the morphology showed no difference when taken in slight ventral flexion or slight retroflexion.) The entire mandible, especially the mandibular angle region, had to be clearly visible on the panoramic radiographs.

### Assessment

**Fig. 1:**
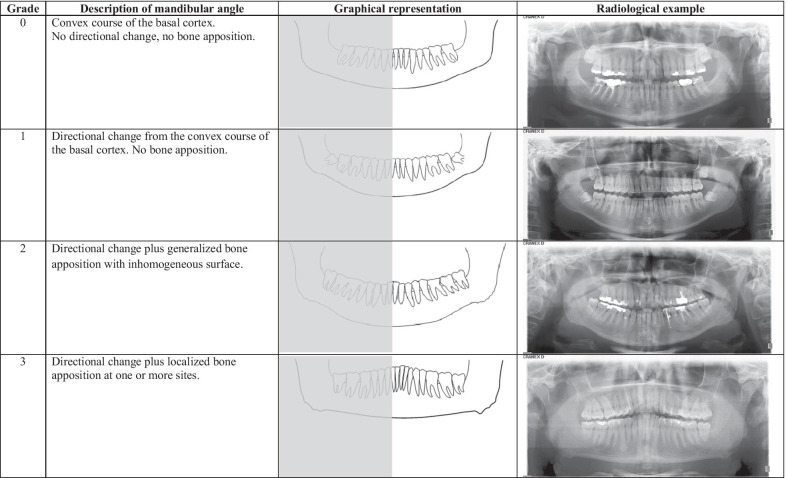
Bone apposition at the mandibular angle and grade classification: grades, description, graphical representation and radiological example

All radiographs were carefully reviewed by two examiners (MS, DD) to obtain an overall impression of the morphology of the mandibular angle. Attention was paid to the recognition of shape patterns of the basal cortical bone in order to classify the observed morphologies into different grades. Subsequently, each of the total 400 mandibular angles was individually assigned to one of the four grades that resulted from the analysis of the radiographic images (Fig. 1). Macroscopically visible bone appositions were differentiated between unilateral and bilateral occurrence.

### Statistical analysis

Odds ratios (ORs), 95% confidence intervals (CIs), and p values were calculated using MedCalc statistical software (Ostend, Belgium). Where zeros caused problems in calculating an OR, the value of 0.5 was added to the corresponding cell of the two-by-two table (Haldane-Anscombe correction [19, 20]).

## Results

Based on the radiological findings, we distinguished four different grades (Fig. 1). Mandibular angles without apposition were assigned to grades 0 or 1 as described in Fig. 1.

Apposition was observed in 95 mandibular angles (47.5%) of 59 adult patients with bruxism (grade 2 and 3). Almost two-thirds of these patients had bilateral appositions, but not necessarily to the same degree (Table 1). With the exception of two mandibular angles, each observed apposition was accompanied by a directional change of the corresponding mandibular angle. In contrast to the bruxism group, none of the adolescents showed bone remodeling. Instead, only grades 0 and 1 (36 and 64 adolescents, respectively) were observed. There were no side differences. The OR for individuals were 288 (95% CI 17–4772; *p* < 0.0001), meaning that an individual diagnosed with bruxism was almost 300 times more likely to show bone apposition than a non-bruxer. The ORs for mandibular angles were 363 (95% CI 22–5904; *p* < 0.0001).

**Table 1:** Distribution of grading in the two groups according to the number of affected individuals and the affected mandibular angles

	Bruxism group		Control group	
	# subjects	# angles	# subjects	# angles
Grade 0	41	29	36	72
Grade 1		76	64	128
Grade 2	59	33	0	0
Grade 3		62	0	0
Bilateral different apposition	8	–	–	–
Bilateral identical apposition	28	–	–	–
Unilateral apposition	23	–	–	–
n	100	200	100	200

In the bruxism group: laterality of apposition

## Discussion

The main findings of our study can be summarized as follows:

1. Bone apposition at the mandibular angles was absent in the adolescents.
2. Conversely, six out of ten bruxers showed apposition.
3. Different degrees of morphological alterations could be distinguished.

These findings may be well explained: It has been reported that functional and parafunctional loading of the mandible generate bending and torsional moments as well as shear forces that lead to tensile and compressive strains and bony deformation [21]. Under such compressive forces, the mandibular angles with their insertions of the masseter and medial pterygoid muscles—among other structures—are particularly stressed [21–24]. For example, using a finite element model Panagiotopoulou et al. [21] showed that when simulating nut chewing very high sagittal shear strains are produced at the lateral surface of the balancing-side mandible [21], at the insertion of the masseter and medial pterygoid muscles.

The control group was composed of adolescents, as bone appositions were not yet expected in this age group. Yet, with an estimated prevalence of sleep bruxism of around 50% in children and adolescents [25], it can be assumed that this behavior was also widespread in the control group. We interpret the nonappearance of bony alterations with the relatively short duration of loading of the mandibular angles, which was apparently insufficient to manifest as visible bone apposition. Of course, one could also conclude that the observed difference in the prevalence of bone apposition is merely an age-effect. However, such an assumption contradicts clinical observations, since such morphological conspicuities are typically seen only on panoramic radiographs of adult bruxers. Nonetheless, to support our opinion, a comparison of age-matched groups of bruxers and non-bruxers would be beneficial to support our hypothesis. Still, identification and selection of the latter is particularly challenging and may require instrumental approaches [13].

The documentation of ventral or retroflexions allowed the assessment of a possible correlation between the fine alignment in the X-ray unit and the visibility of the bone appositions. Since studies of panoramic radiographs in slight ventral flexion and panoramic radiographs in slight retroflexion yielded the same results, some flexion during imaging may be considered irrelevant. The exact location of the appositions (laterally or medially) could not be defined due to the summation effect of panoramic radiographs, but this was irrelevant in our study.

## Conclusions

In addition to self-report and clinical examination, radiologically diagnosed bone apposition may serve as an additional diagnostic indicator of bruxism. Since such morphological formations require a relatively long developmental period (many years), they may provide an indication regarding the presence of bruxism that has existed for a long time. This additional information may be helpful when communicating with patients, especially since they are often unaware of the existence of this parafunction.

## Data Availability

The datasets used and/or analyzed during this study belongs to the authors and are available from the corresponding author only upon on reasonable request.

## Abbreviations

CI: Confidence interval
N: Newton
OR: Odds ratio
TMJ: Temporomandibular joint
